# Caspase-8 Binding to Cardiolipin in Giant Unilamellar Vesicles Provides a Functional Docking Platform for Bid

**DOI:** 10.1371/journal.pone.0055250

**Published:** 2013-02-13

**Authors:** Olivier Jalmar, Liberty François-Moutal, Ana-Jesus García-Sáez, Mark Perry, Thierry Granjon, François Gonzalvez, Eyal Gottlieb, Jesus Ayala-Sanmartin, Beate Klösgen, Petra Schwille, Patrice X. Petit

**Affiliations:** 1 Université Paris-Descartes, Centre de recherche des Saint-Pères, UMR S-747, “Toxicology, Pharmacologie et Signalisation cellulaire”, Paris, France; 2 Institut de Chimie et Biochimie Moléculaires et Supramoléculaires, UMR CNRS 5246 - ICBMS, Villeurbanne, France; 3 Biophysics Group, BIOTEC, TU Dresden, Dresden, Germany; 4 Department of Physics, Chemistry and Pharmacy, MEMPHYS - Center for Biomembrane Physics, University of Southern Denmark, Odense, Denmark; 5 Cancer Research UK, The Beatson Institute, Glasgow, Scotland, United Kingdom; 6 CNRS, UMR 7203, Laboratoire des Biomolécules, Ecole Normale Supérieure, Paris, France; Karolinska Institutet, Sweden

## Abstract

Caspase-8 is involved in death receptor-mediated apoptosis in type II cells, the proapoptotic programme of which is triggered by truncated Bid. Indeed, caspase-8 and Bid are the known intermediates of this signalling pathway. Cardiolipin has been shown to provide an anchor and an essential activating platform for caspase-8 at the mitochondrial membrane surface. Destabilisation of this platform alters receptor-mediated apoptosis in diseases such as Barth Syndrome, which is characterised by the presence of immature cardiolipin which does not allow caspase-8 binding. We used a simplified *in vitro* system that mimics contact sites and/or cardiolipin-enriched microdomains at the outer mitochondrial surface in which the platform consisting of caspase-8, Bid and cardiolipin was reconstituted in giant unilamellar vesicles. We analysed these vesicles by flow cytometry and confirm previous results that demonstrate the requirement for intact mature cardiolipin for caspase-8 activation and Bid binding and cleavage. We also used confocal microscopy to visualise the rupture of the vesicles and their revesiculation at smaller sizes due to alteration of the curvature following caspase-8 and Bid binding. Biophysical approaches, including Laurdan fluorescence and rupture/tension measurements, were used to determine the ability of these three components (cardiolipin, caspase-8 and Bid) to fulfil the minimal requirements for the formation and function of the platform at the mitochondrial membrane. Our results shed light on the active functional role of cardiolipin, bridging the gap between death receptors and mitochondria.

## Introduction

The initiation of apoptosis leads to distinct morphological changes culminating in the dismantling of the cell by a family of cysteine proteases called caspases [1] and ultimate cell clearance by other cells. Apoptosis can proceed by either the intrinsic or the extrinsic pathway [2]. CD95 (APO-1/Fas) has become the model death domain-containing receptor, and it is the most extensively studied death receptor that activates the extrinsic apoptosis pathway. The triggering of this receptor results in the formation of the death-inducing signalling complex (DISC), a complex of signalling proteins recruited by activated CD95 immediately after the addition of agonistic anti-CD95 antibodies or the CD95 ligand [3]. The formation of the DISC is associated with the recruitment and activation of caspase-8 and the direct cleavage of downstream effector caspases. The formation of the DISC, consisting of the adapter molecule FADD/MORT1 [4], [5] and caspase-8 [6], [7], [8] results in the release of active caspase-8 at the DISC and the cleavage of various intracellular death substrates [9], [10]. The DISC proteins, FADD and caspase-8, have been shown to be essential components of the CD95 signalling machinery [8], [11], [12], [13]. In contrast, the intrinsic apoptosis pathway is triggered from within the cell, either by the direct activation of caspases or through intracellular changes, such as DNA damage, which result in the release of pro-apoptotic factors and the activation of effector caspases.

**Figure 1:**
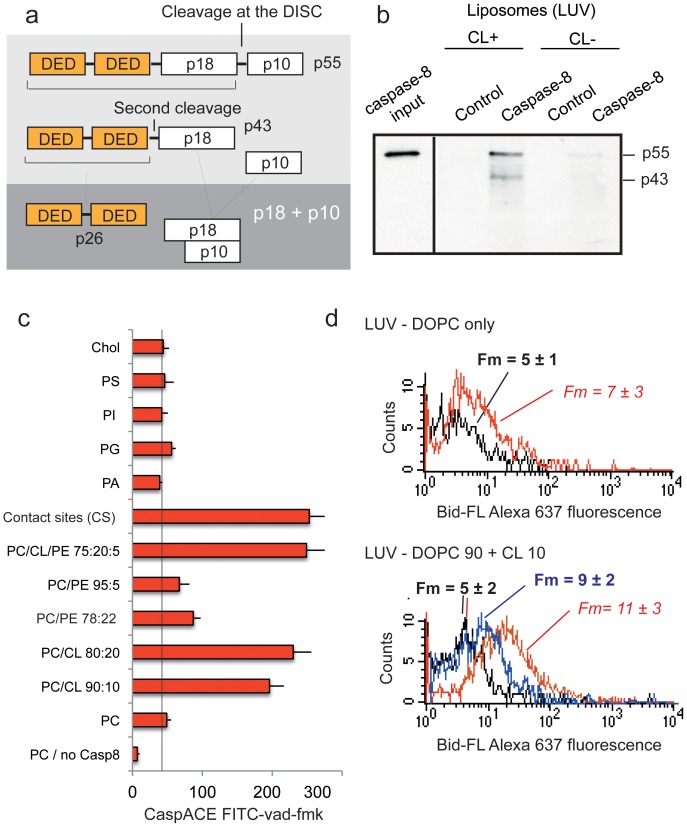
Binding of Bid and caspase-8 to CL-containing large unilamellar liposomes (LUVs). (**a**) Schematic diagram of caspase-8 autoprocessing during Fas-mediated apoptosis. Upon dimerisation, procaspase-8 (p55) is initially cleaved between its two active subunits, p18 and p10, to generate the p43/p10 heterodimer; p43 is then cleaved between the death effector domain (DED) and the p18 subunit, to produce the fully active p18/p10 form. (**b**) Western blot analysis of caspase-8 binding to the “contact site mimetic” liposomes or similar liposomes without CL, in which the CL was replaced with PE (22%) (**c**) Caspase-8 binding, as detected by caspACE FITC-VAD-fmk binding to the active site, to liposomes of various compositions (monolipid liposomes made from PA, PC, PE, PI, PG or cholesterol, and mixed liposomes composed of DOPC+CL, DOPC+PE, DOPC+CL+PE at various molar ratios, contact site mimetic liposomes; for details see materials and methods). (**d**) Flow cytometric analysis of CL^+^ and DOPC-only liposomes in the presence or absence of Bid_Alexa488_. The black spectrum correspond to control vesicles whereas the red spectrum correspond to the vesicles plus Bid_Alexa488_. The blue spectrum results from an alkaline wash of the CL^+^ liposomes. The alkaline wash involved centrifugation of liposomes and resuspending them in 0.1 M Na_2_CO_3_, pH 11.5. The liposomes were then analysed directly by flow cytometry. Fm: fluorescence mean value, in arbitrary units (a.u.).

In the death receptor pathway of apoptosis induction, the best characterised connection between the two pathways is Bid, a member of the Bcl-2 family that is translocated to the mitochondria after cleavage by caspase-8. The dimerisation of two caspase-8 monomers (p55/p55) results in a conformational change that exposes the active site of the caspase through a mechanism known as ‘induced proximity’ [14], [15]. Dimerisation was shown to be sufficient for the activation of caspase-8, but it has been suggested that full activity may require self-cleavage [14], [16], [17], [18]. Caspase-8 initially cleaves itself between the p18 and p10 domains, forming a heterodimer within a heterotetrameric complex (p43–p10/p43–p10) (**Fig. 1a**). This first cleavage is necessary for the recognition of other substrates, including effector caspases (such as caspase-3) and the pro-apoptotic Bcl-2 family member Bid [16], [17]. Extrinsic apoptosis follows one of two pathways, type I or type II, depending on the level of caspase-8 activation upon DISC formation [7]. In the type I pathway, large amounts of DISC and active caspase-8 are formed, leading to the direct cleavage of effector caspases in the cytosol [19]. In the type II pathway, DISC assembly is slower, and smaller amounts of active caspase-8 are generated [7]. XIAP (X-linked inhibitor of apoptosis) was shown also to inhibit this pathway [20]. Thus, cells containing large amounts of XIAP require a tBid mitochondrion-mediated amplification of the caspase cascade to overcome the caspase inhibition by XIAP. In this context, caspase-8 must be engaged in the intrinsic pathway to amplify the death signal and execute apoptosis. Transition from the extrinsic pathway to the intrinsic pathway is achieved through the processing of Bid by caspase-8 [21], [22], leading to the generation of tBid, which then interacts with cardiolipin via its hairpin-forming domain [23]. This interaction disturbs mitochondrial bioenergetics, leading to Bax/Bak delocalisation [24] and permeabilisation of the mitochondrial outer membrane (MOMP).

We recently showed that the mitochondrial surface becomes enriched in caspase-8 during type II extrinsic apoptosis induced by Fas. Proof of this concept was obtained with lymphoblastoid cells (type II cells) derived from Barth syndrome patients and tafazzin knock-down HeLa cells, which contain no mature cardiolipin (CL) but large amounts of monolysocardiolipin [25]. We also showed that a blockade of the association of caspase-8 with mitochondria due to cardiolipin deficiency resulted in the inhibition of p43–p10 formation, preventing both Bid cleavage and apoptosis [25]. It has also recently been shown that caspase-8 and Bid form a supramolecular complex on the surface of the mitochondrial outer membrane [26], in so-called “mitosomes”. There is thus a mechanism by which low levels of proteolytically active caspase-8 can specifically target sufficient amounts of Bid at the surface of mitochondria, to produce tBid [26]. It was also shown that tBid binds CL [23], [24], [27], [28]. Thus, contact sites between the inner and outer mitochondrial membranes are enriched in CL, which is predominantly found in the inner mitochondrial membrane and can adopt an H_II_ conformation [29], rendering it accessible from outside the mitochondria. This location provides CL with access to all the factors required for the formation of a caspase-8/cardiolipin/Bid platform at the mitochondrial membrane surface.

Confirmation of a key role for CL in platform formation requires investigation of the basic components of this platform in a simplified “*in vitro”* system, thus avoiding confounding effects of unknown factors. Cell-free model systems have been shown to reproduce correctly the behaviour of Bcl-2 proteins during apoptosis [30], [31], [32], [33]. In this study, we developed a simplified system constituted of giant unilamellar membranes, with or without CL, to investigate interactions between caspase-8 and CL and to seek experimental evidence for the functional activity of the caspase-8/Bid/cardiolipin platform. Our findings shed light on a fundamental aspect of cell death-activating processes and especially on the major role of cardiolipin in both the formation and functional activity of the reaction platform. Our data are consistent with a model in which caspase-8 binding to CL is a key step in early apoptotic signal transduction, linking the Fas-receptor complex with mitochondria. This model suggests that lipid/protein interactions at the mitochondrial membrane are of major importance and unravels the “embedded together” model of the interaction of Bcl-2 family members with intracellular membranes.

## Experimental Procedures

All the fluorescent probes were from Molecular probes (Invitrogen, Life Technologies SA, Saint-Aubin, France) and all lipids were from Avanti Polar Lipids, Inc. (Alabaster, Alabama, USA).

### Protein Preparation

**Figure 2:**
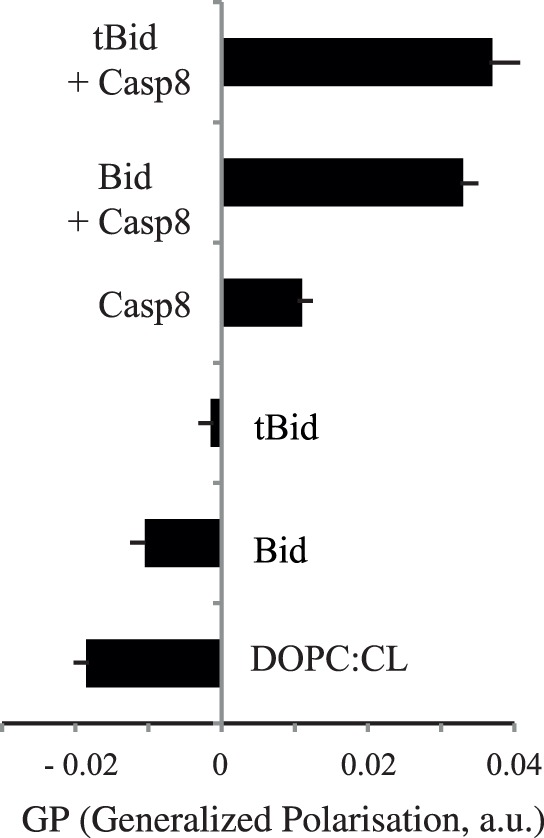
Analysis of the effects of caspase-8, Bid and tBid on the Laurdan fluorescence of CL^+^ and CL^−^ liposomes. Generalised polarisation (GP, arbitrary units, a.u.) determined from Laurdan fluorescence measurements. GP values are reported for the various preparations, as described in the materials and methods.

We prepared fluorescently labelled human tBid, as previously described [34], from Bid cDNA in pET15b with a single cysteine residue at position 64 in the tBid fragment, which we labelled with Alexa647 maleimide (Invitrogen) [33]. We purified full-length Bid (Bid) with the same protocol as for tBid, but from a cDNA with C15S and C28S mutations and with Bodipy488 or Alexa647 maleimide labelling. Bid and non-fluorescent tBid were kindly provided by J.C. Martinou (Geneva, Switzerland). For the caspase-8 we have used two sources: 1 - An in vitro translated P_55_ form purified as described in the work of Gonzalvez et al. [25] (essentially used for the work with liposomes in Figure 1) and 2 - The second caspase-8 has been provided by J.C. Martinou (P_10_ and P_18_ subunits expressed separately in Escherichia Coli and reconstituted in their active form). This is what was used in the article unless indicated otherwise.

### Preparation of Liposomes

All liposomes were prepared as previously described [27]. Liposomes contained either the same lipids as mitochondrial contact sites (CS) - 9% cholesterol, 22% phosphatidylethanolamine (PE), 8% phosphatidylinositol (PI), 20% cardiolipin (CL) and 34% phosphatidylcholine (PC) [35] - or each individual lipid from CS, tested separately and with PC used to make up the difference. The lipid ratios in the so-called single lipid liposomes were as follows: PC, 100% PC; PI, 8% PI and 92% PC; Chol, 9% cholesterol and 91% PC; PE, 22% PE and 78% PC; PG, 20% phosphatidylglycerol and 80% PC; PA, 20% phosphatidic acid and 80% PC. PC/no Casp8 are PC liposomes with no addition of caspase-8. For all PC liposomes containing CL and PE, the corresponding proportions are indicated on the graphs. The liposomes, suspended in light buffer (100 mM NaCl, 2 mM MgCl_2_ and 10 mM Tris-HCl, pH 7.1), were centrifuged for 1 h at 55,000 rpm in a Beckman SW 70.1 rotor at 10°C.

### Binding of Bid and Caspase-8 to Liposomes

Pelleted liposomes were obtained as described above (the liposome mixture was shiny and free of aggregates); 50 µl of the precipitate was resuspended in 450 µl of light buffer and incubated with either caspase-8 (290 nM) or Bid (50 nM) for flow cytometry analysis.

### Immunoblot Analysis

The liposome pellet was lysed in 1% sodium cholate, resuspended in a protein sample buffer containing DTT, and resolved by SDS-PAGE in a 4–12% polyacrylamide gel (NuPAGE). The proteins were transferred to a membrane and p55 and p43 bands were detected with anti-caspase antibodies directed against the DED domain of caspase 8 (Becton-Dickinson).

### Preparation of Giant Unilamellar Vesicles

The GUVs consisted mostly of DOPC and CL, with CL content ranging from 0 to 20% (mol/mol), as indicated in the figure legends. All lipid mixtures were prepared in chloroform stock solution, at a total concentration of 1 mg/ml, with the appropriate lipid DOPC/CL ratio. Vesicles were grown in sucrose solutions (300 mOsm). For confocal microscopy, GUVs were prepared by the electro­swelling method [36]. We spread 5 µl of lipid mixture (1 mg ml^−1^ in chloroform) directly onto two Pt wire electrodes kept 1 cm apart in a swelling chamber. The chamber was filled with swelling solution (300 mM sucrose) and the wires were connected to a power generator; a voltage of 2.3 V at 10 Hz was applied for 1 h at room temperature, for the field-supported swelling of GUVs from the lipid films. The GUVs were then detached from the electrodes by increasing the frequency to 2 kHz for 30 min. Finally, they were carefully harvested with a syringe with a large-diameter needle.

For flow cytometry, GUVs were prepared by the electroformation technique, from lipid films deposited on ITO slides [37].

### Laurdan Fluorescence Measurements

Generalised polarisation experiments were carried out with Laurdan, as follows: Laurdan was added to the phospholipid solution in chloroform such that the molar ratio of dye to lipid was 400∶1. The solvent was removed by evaporation and the dry lipid film was hydrated (20 mg/ml) by incubation in phosphate citrate buffer (pH 7.0). The liposomes were then prepared as previously described [38]. Fluorescence was measured in a Hitachi F4500 fluorometer (150 W Xe). A band-pass setting of 2.5 nm was used for both excitation and emission. Liposomes were incubated with proteins for 1 hour and then centrifuged at 160,000 g, for 1 hour in an Airfuge centrifuge. Spectra were recorded for the resuspended pellets in a thermostatically controlled quartz cuvette (1 cm path length). We recorded 3D spectra with the following parameters: excitation wavelength from 320 to 420 nm (1 nm gap) and emission wavelength from 420 to 550 nm, at 37°C, on 150 µg Laurdan liposomes in the presence of 1 nM tBid or 50 nM Bid and/or 290 nM procaspase-8. The excitation generalised polarisation was calculated as previously described [39]: 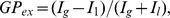
where I_g_ and I_l_ are the fluorescence intensities at the maximum emission wavelength in the gel and in the liquid crystalline phases, respectively, at a fixed excitation wavelength (360 nm).

### Microaspiration Studies

The mechanical response of test membranes to CL and tBid was studied in microaspiration experiments, which were carried out and analysed as previously described [40]. Isolated single GUVs swollen in 300 mM sucrose (CL/DOPC = 5%) and transferred to iso-osmolar glucose solution for contrast enhancement were exposed to an increasing membrane tension by microaspiration. A series of snapshots taken from a video recording at various aspiration pressures [600 Pa, 1600 Pa] was analysed for each GUV, to obtain the expansion modulus K_s_ (mN/m) and the rupture tension τ_r_ (mN/m) for the recorded data. The results are expressed as the means for several isolated vesicles studied under conditions that are as close to identical as possible.

### Confocal Microscopy

We resuspended 50 µl of GUVs electroswollen in 300 mM sucrose in 500 µl PBS containing the following proteins: 9 nM Bid Bodipy_488_ and/or 290 nM unlabelled procaspase-8. Caspase-8 and Bid, in the presence of caspase-8, bound very rapidly, so measurements were made immediately, at room temperature. We used a LSM 510 Meta microscope (Zeiss) with a 40× 1.2 NA C-Apochromat water objective (Zeiss) in multitrack mode. We used UV/488/543/633 and 545 nm filters as the principal and secondary dichroic filters. We used an argon laser operating at an excitation wavelength of 488 nm, with a 505–530-nm band-pass filter for the green channel, whereas a red diode laser operating at an excitation wavelength of 633 nm, with a 650-nm long-pass filter for the red channel. The DiD [(1,1′-dioctadecyl-3,3,3′,3′-tetramethylindodicarbocyanine, 4-chlorobenzenesulfonate salt (‘DiD’ solid)] used to stain the lipid in the GUV was from molecular probes (InVitrogen, USA). Images were processed with ImageJ software (http://rsbweb.nih.gov/ij/).

### Flow Cytometry Analysis

Aliquots of 10 µl of a solution of electroformed GUVs in 100 µl of PBS was made up to a volume of 500 µl with PBS for flow cytometry analysis, which was carried out as previously described [41]. When necessary, GUVs were incubated with sedimented proteins and the washed pellet, to eliminate the non-specific binding of dyes and proteins, before flow cytometry. Most experiments were performed online in the flow cytometer: the reaction was started by adding the proteins directly to the tube during data recording, and protein binding and enzymatic activity were detected by monitoring changes in light scattering or by fluorescence measurements. We added Bid-Alexa_647_ to concentrations ranging from 10 to 100 nM. Caspase-8 was added to a concentration of 290 nM. We used a FACS Calibur 4C (Becton-Dickinson) machine equipped with an argon laser operating at 488 nm and a red diode laser operating at 635 nm. A 530±30 nm band pass filter was used for green fluorescence and a 661±16 nm bandpass filter was used for red fluorescence. Beads (10 µm) were added to the samples for use as size markers, when appropriate.

## Results

We tested for direct interaction between caspase-8 and cardiolipin, by incubating liposomes with the same lipid composition as the mitochondrial contact site prepared as previously described [28] with *in vitro*-translated caspase-8 (p55). Western blot analysis of the precipitated liposomes (**Fig. 1b**) showed that the intensities of the p43–p55 caspase-8 bands were significantly stronger in the presence of CL than in its absence, with a greater abundance of the p43 form. In samples from CL-deficient liposomes these two bands were barely detectable (about 45 to 55% of the total caspase-8 loaded onto the gel bound to liposomes). This significant enrichment in the p43-processed form of caspase-8 may be due to the activation of caspase-8 on the liposome or the higher affinity of cleaved p43 for the membrane. We investigated the function of CL in the binding of caspase-8 to liposomes further, using different liposome compositions ranging from single-lipid combinations of phosphatidylcholine (PC) and CL to liposomes mimicking mitochondrial contact sites [27]. Caspase-8 clearly showed a marked tendency to bind to CL-containing liposomes, whereas phosphatidylethanolamine (PE) liposomes bound caspase-8 only weakly (**Fig. 1c**). Bid showed no specific binding to DOPC-only or CL^+^-LUVs; however, low levels of binding to the contact sites of mimetic liposomes (see materials and methods) were observed. Moreover, washing the liposomes in an alkaline solution before flow cytometry analysis dissociated most of the Bid from the CL^+^-LUV (**Fig. 1d**). The very small amounts of Bid present on LUVs may therefore be attributed purely to non-specific binding.

### Changes in Liposome Membrane Fluidity Due to Successive Binding to Caspase-8 and Bid

The fluorescence properties of Laurdan were used to monitor fluctuations, due to protein binding, in the organisation and fluidity of the surrounding lipid membrane. Generalised polarisation (GP; as presented in the materials and methods section) was measured on liposomes consisting of either DOPC or a mixture of DOPC and CL, after the separate or simultaneous addition of procaspase-8 and Bid or tBid (**Fig. 2**). The data obtained indicate that a low GP value was associated with high fluidity of the “DOPC-CL”-system and that this property was not significantly modified by the addition of Bid. Indeed, Bid had only a small effect on the GP of DOPC-CL vesicles, whereas the addition of caspase-8 was followed by an increase in the GP. tBid alone modified the GP considerably and the combination of Bid and caspase-8 gave values similar to those obtained with tBid and caspase-8.

### Tension/rupture of Cardiolipin-containing Giant Unilamellar Vesicles (GUVs) in the Presence or Absence of Caspase-8 and Bid Proteins

The effects of caspase-8, caspase-8+ Bid and/or tBid on membrane fluidity in Laurdan experiments (**Fig. 2**) suggest that the interaction of these proteins with a target membrane affects the elastic properties of the membrane. The elasticity theory for membranes is based on the theory of thin liquid films [42]. Two basic deformations can be identified: bending perpendicular to the bilayer surface, described by a bending modulus κ, and lateral membrane compression/expansion within the bilayer plane, quantified as a lateral compression modulus K_s_ [43]. Membrane elasticity depends on the composition of the bilayer and its thermodynamic state (gel/liquid) [44]. In pure lipid membranes, it reflects interactions within the bilayer (lipid:lipid), the details of which are modified by the presence of any other molecules within, or along the aqueous interface of, the bilayer [40]. Thus, membrane elasticity responds to the insertion/adsorption of foreign molecules, in turn potentially affecting the association affinities of these molecules [45], [46] or their function within the membrane [47], [48], [49].

We investigated the interaction and its mechanical effects, by developing a minimal giant unilamellar vesicle (GUV) model membrane system consisting of DOPC and DOPC-CL (95/5; mol/mol). This model, although highly simplified, may be considered to mimic mitochondrial contact sites. The micropipette aspiration technique was used to explore the effects of the presence of CL on the mechanical properties of the DOPC host membrane and of the apoptotic proteins tBid and caspase-8. The area expansion modulus (K_s_), and the lysis tension (or tensile breaking strength, τ_r_) were used to quantify membrane stability.

**Figure 3:**
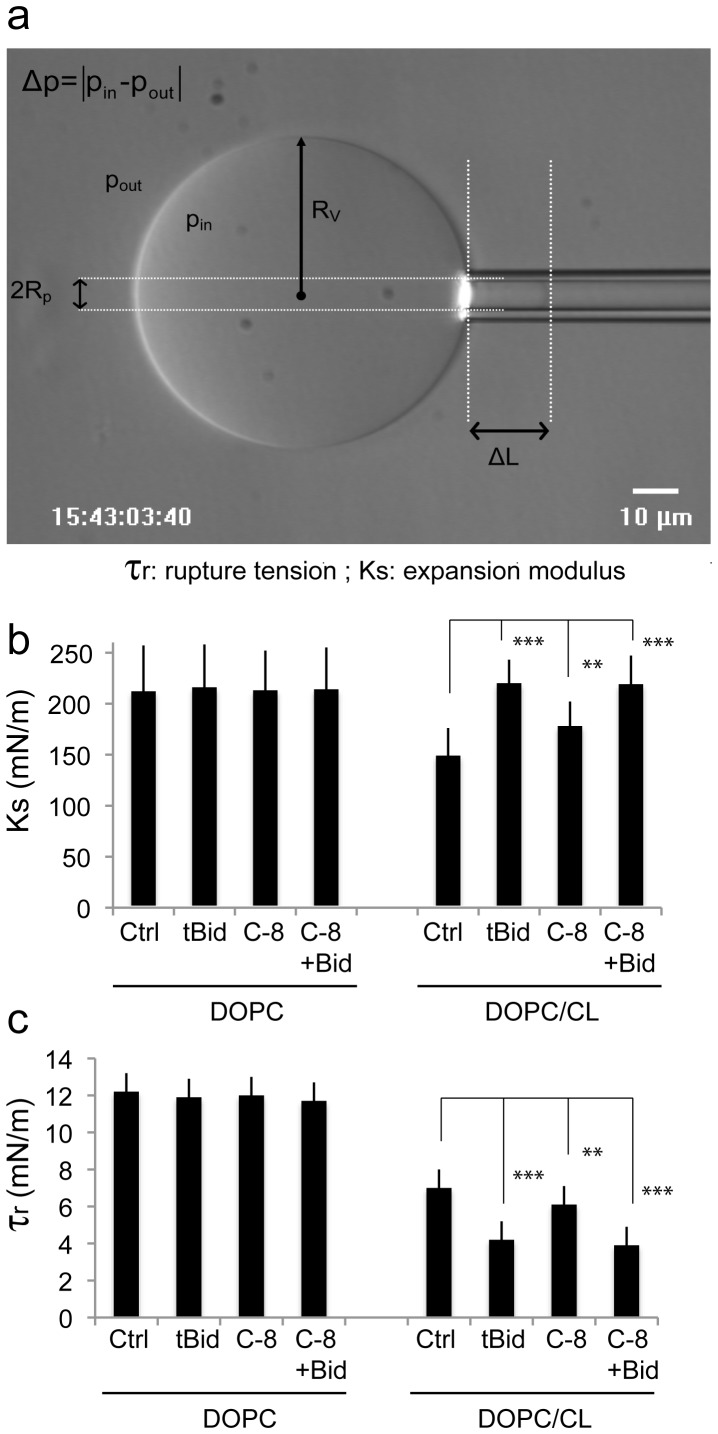
Determination of the micromechanical properties of giant unilamellar vesicles (GUVs) by microaspiration. (a) Video micrograph of a vesicle aspirated in a glass suction capillary. The principal variables for the determination of the area expansion modulus are indicated: R_V_: vesicle radius, p_in_ and p_out_: pressure inside and outside the vesicle, ΔL: length of membrane meniscus inside a glass pipette of internal radius R_p_. Excess membrane tension τ is created by suction such that Δp≠0. (b and c) Histograms of the micromechanical quantities measured in the test system under various experimental conditions. (b) K_s_: expansion modulus (mN/m); (c) τ_r_ : tensile breaking strength (mN/m). Caspase-8 was added to a final concentration of 290 nM, tBid to 30 nM and Bid to 50 nM. Fisher’s test were used for statistical analyses of differences for both K_s_ and τ_r_ measurements (**, p<0.01 and *** p<0.05).

The principal set-up and some results, compiled into two histograms, are shown in **Fig. 3** **.** The simple presence of any of the proteins investigated - tBid, caspase-8, and caspase-8 with Bid - had no effect on the mechanical stability of the DOPC test membrane. On the contrary, the addition of CL to a DOPC host membrane had a clear impact: it decreased the area expansion modulus and strongly decreased the overall sustainability of the membrane when subjected to mechanical stress, as demonstrated by the low value of the rupture tension τ_r_. In the presence of tBid, the membrane stability, assessed as the expansion modulus K_s_, returned to its initial value (**Fig. 3b**), but there was a further decrease in the rupture tension τ_r_ (**Fig. 3c** **)**, to about 30% of the value initially obtained for the pure DOPC reference membrane. The simultaneous presence of caspase-8 and Bid resulted in similar values, whereas the addition of caspase-8 alone gave intermediate values.

### Confocal Microscopy Investigations of the Various Proteins Binding to GUVs

**Figure 4:**
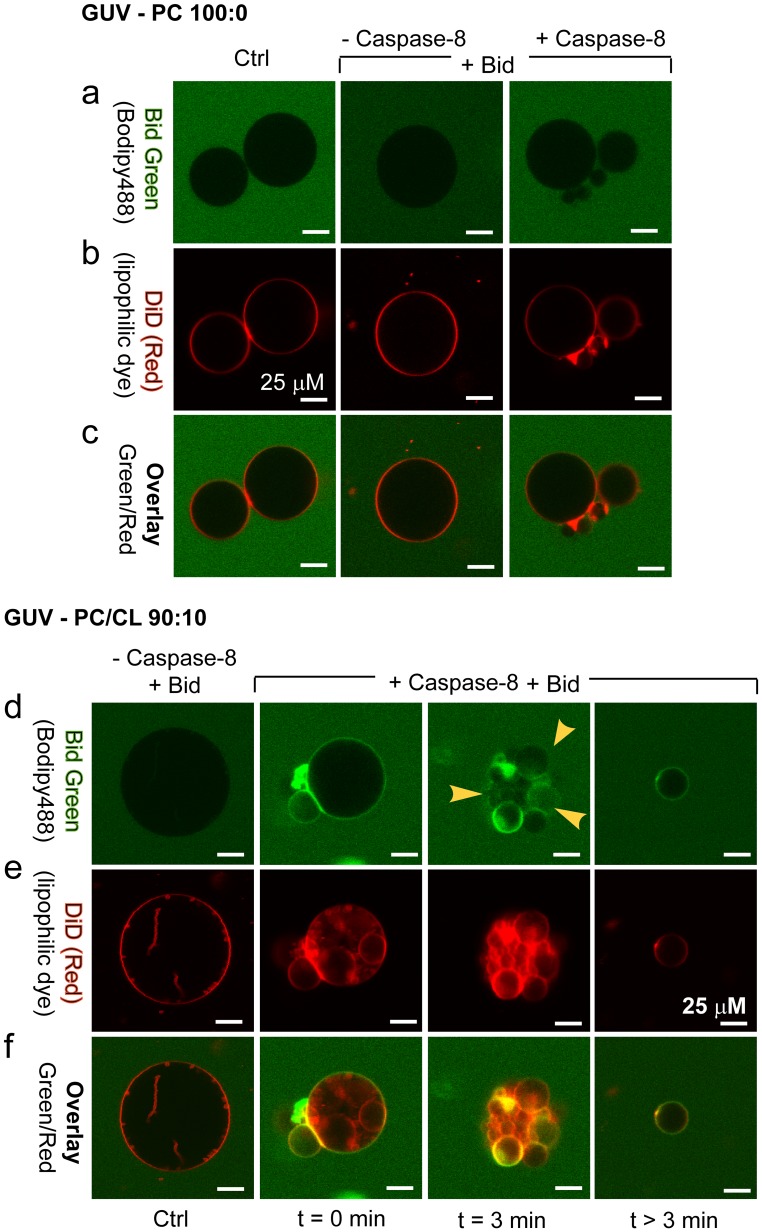
Confocal microscopy study of the binding of Bid and caspase-8 to giant unilamellar vesicles containing cardiolipin. Trios of images (top, middle and bottom) for the same sample: two images obtained with two different detector channels of the microscope, together with an overlay image. DOPC-only (100∶0) vesicles are presented in panels **a** to **c** and DOPC/CL (90∶10) vesicles in panels **d** to **f**. Top: in **a** and **d**, protein binding to GUVs shown in green (this binding only becomes apparent when the green label accumulates at the membrane); middle: the GUV membrane was labelled with 0.05% of the hydrophobic dye DiO, as shown in (**b, c**) and in red, as shown in (**e, f**); bottom: overlay of green and red images (**c, f**). Time is indicated in minutes. The arrows indicate the decrease in GUV fluorescence following the formation of a complex between procaspase-8 and Bid_Alexa488_, resulting in a non-fluorescent tBid.

Confocal microscopy provided evidence of an interaction between proteins and the test membranes. A multicolour approach was used: the membrane was labelled with DiD, shown in red in **Fig. 4** **,** with Bid shown in green. The images show the results of staining with the two dyes individually and the simultaneously obtained overlay image. Bid_Green_ did not bind to vesicles containing phosphatidylcholine alone (**Fig. 4a**) or to CL-containing vesicles (not shown). These results are consistent with previous reports that Bid does not bind to either DOPC or DOPC/CL GUVs [33]. Bid_Green_ did not bind to DOPC vesicles after the addition of caspase-8 (**Fig. 4a**), unless CL was also present (**Fig. 4d**). The binding of Bid_Green_ to vesicles thus appeared to require the presence of both CL within the membrane and caspase-8 binding to it (**Fig. 4d**). The short-term effects of caspase-8/Bid_Green_ on CL-GUVs included not only complex binding (**Fig. 4d**), but also vesicle reorganisation and collapse (**Fig. 4d–f**). The vesicles also displayed a significant decrease in green fluorescence (**Fig. 4d**) within a few minutes of addition of Bid_Green_. This decrease in fluorescence resulted from cleavage of the tagged BH1–BH2 domain, the fluorophore remaining in the soluble p7 part of the protein after cleavage by capase-8 (as illustrated in **Table 1** **)**. These observations provide evidence of a reaction platform, consisting of CL/caspase-8 and Bid, presenting an enzymatic activity. The CL-containing GUVs had a low rupture tension (**Fig. 3c**); they frequently broke and resealed, forming either smaller vesicles or, more often, irregularly shaped aggregates, due to the defective reorganisation of membrane material.

**Table 1:** Flow cytometry analysis of the GUVs.

Conditions	Mean fluorescence (arbitray units, a.u.)
GUVs control	7±3
GUVs+Caspase-8	8±3
GUVs+Caspase-8+ Bid (0 min)	84±15
GUVs+Caspase-8+ Bid (3 min) P1 (10%)	232±50
GUVs+Caspase-8+ Bid (3 min) P2 (90%)	45±12
GUVs+Caspase-8+ Bid (>3 min)	19±5

The caspase-8+ Bid binds immediately to the GUVs when they contain CL. At 3 min, the caspase-8/bid system is functional and two subpopulations of vesicles are present: one with a higher fluorescence (P1, 10% of the vesicles) and one with lower fluorescence (P2, 90% of the vesicles). After 3 min, the vesicle are small and exhibit weak fluorescence indicating the loss of p7 fluorescence resulting from the full cleavage of Bid (fluorescent) to tBid (non fluorescent).

### Flow Cytometry Analysis of the Functional Activity of the Caspase-8/Bid/cardiolipin Platform

**Figure 5:**
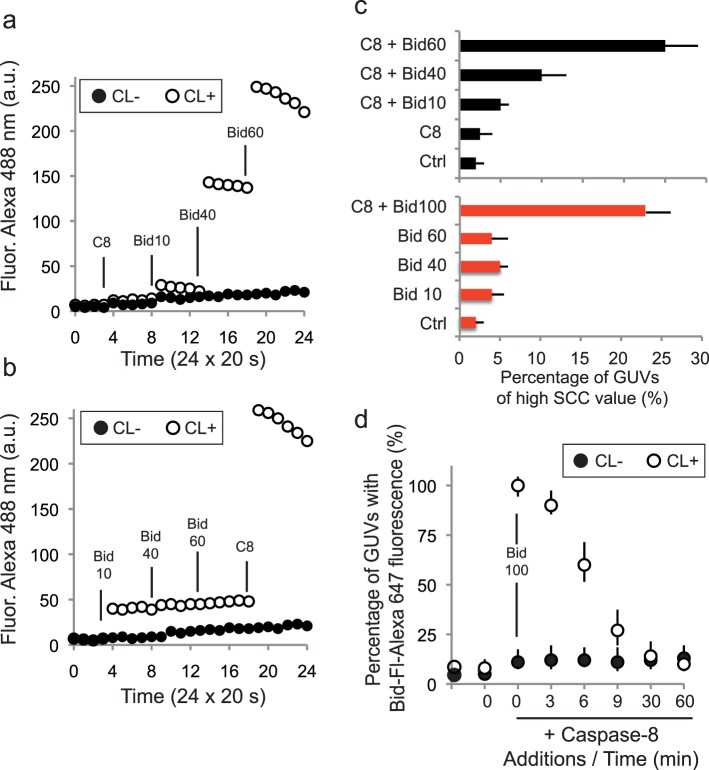
Flow cytometric analysis of the interaction between CL-GUVs and caspase-8-Bid. (**a–b**) Short-term effects (20 time points at 20 s intervals, total 6.66 min) of successive additions of procaspase-8 or Bid to GUVs-CL. (**a–b**) Each product (caspase-8 or Bid) was added progressively, at 1.66-minute intervals, as shown in the recording, and the mean fluorescence of the vesicles was then measured. (**a**) Caspase-8 (Casp8) was added before Bid whereas, in (**b**), caspase-8 was added after three successive additions of Bid (10 nM, 40 nM and 60 nM). Even shortly after additions, the enzymatic system was functional, provided that caspase-8 bound to the giant unilamelar liposomes (GUVs). (**c**) the upper histogram, in black, corresponds to (**a**), and the lower histogram, in red, corresponds to (**b**); the occurrence of vesicles with a higher side scatter (SSC), due to procaspase-8/Bid cleavage activity, was recorded and is plotted as a percentage (%) of the total vesicle population. (**d**) The intensity of Bid-Alexa_647_ fluorescence associated with GUVs is shown as a function of procaspase-8 addition and time, for GUVs with (closed circles) or without (open circles) CL.

Flow cytometry has rarely been used to follow and characterise giant unilamellar vesicles interacting with proteins [50], [51]. We recently reported a flow cytometry analysis of giant unilamellar vesicles based on both their light scattering and fluorescence properties [41]. CL-GUVs were analysed after the addition of protein and their fluorescence was recorded during the initial stages of the interaction (**Fig. 5a**). In the presence of caspase-8, vesicle fluorescence was detected immediately after the first addition of Bid (**Fig. 5a**). The subsequent addition of larger amounts of Bid-Alexa_488_ enhanced this fluorescence. In the absence of caspase-8, the increase in fluorescence was not significant (**Fig. 5b**). However, membrane-associated Bid fluorescence increased immediately after the addition of caspase-8 to the system.

This binding was also associated with a change in the light-scattering properties of the vesicles (**Fig. 5c**), evidencing a change in vesicle size or shape distribution. Indeed, as described above (**Fig. 4** **)**, the binding of caspase-8 and Bid resulted in the disruption of CL-containing GUVs to form aggregates and smaller vesicles. In the absence of caspase-8, no change in side scatter was recorded (**Fig. 5c**, lower histogram): the initially injected vesicles were stable. We followed changes in the number of Bid-labelled vesicles over time (**Fig. 5d** **)**. After the addition of caspase-8, all CL-containing GUVs were labelled with fluorescent Bid (closed circles). The number of Bid-labelled vesicles decreased over time, due to the cleavage of the Alexa_488_-labelled Bid domain after caspase-8 action. The large decrease in vesicle fluorescence provides further evidence for the activity of the newly formed caspase-8-Bid-CL platforms. Pre-incubation of the system with general caspase inhibitors (z-VAD-fmk and Boc-D-fmk) or a specific caspase-8 inhibitor (z-IETD-fmk) abolished the fluorescence drop due to Bid cleavage by caspase-8 (not shown).

## Discussion

Caspase-8 interacts with mitochondria in both healthy [52] and apoptotic [53], [54] cells. However, it has remained unclear how caspase-8 interacts with CL in mitochondria. It has been suggested that this enzyme is translocated into mitochondria together with its known substrate, Bid [25]. However, caspase-8 translocation to the mitochondria after Fas activation is unaffected in Bid knock-down cells. Caspase-8 interaction with mitochondria may be mediated by other proteins [54] or, as described for tBid, caspase-8 may interact not only with other proteins, but also directly with the lipid CL at the mitochondrial membrane. The “embedded together” model for the association of Bcl-2 family members with the lipid domain of membranes assumes that the insertion of these proteins into the mitochondrial outer membrane during apoptosis affects the affinities of the various Bcl-2 proteins, creating new interaction surfaces [30], [55]. It has been conjectured that mitochondrial-membrane microdomains enriched in CL play an important role in apoptosis and enzyme flux control [56].

We investigated the role of CL in the formation of such an apoptosis-activating reaction platform, by generating a minimal *in vitro* reconstitution system with biomimetic membranes (LUVs and GUVs). Western blotting and flow cytometry (**Fig. 1**) were used to distinguish between the specific and non-specific binding of Bid and caspase-8. Indeed, whereas Bid interacted with neither DOPC-only nor CL^+^-liposomes, caspase-8 was found to interact with CL-containing LUVs, giving rise to the p43 kDa CL-activated form (**Fig. 1b, c**).

We then used Laurdan as a fluidity tracer, to study the effects of caspase-8, Bid and the caspase-8+ Bid complex on a relevant membrane model. Differences in the excitation and emission fluorescence spectra of Laurdan in the gel and liquid-crystalline phase make it possible to use the general polarization (GP) parameter to report on the local changes in membrane water content related to changes in membrane fluidity due to protein binding. Bid alone did not bind to liposomes (**Fig. 4**). By contrast, caspase-8 and caspase-8 plus Bid decreased the fluidity of CL-containing membranes (**Fig. 2**), as, to a lesser extent, did tBid. This result is consistent with previous data indicating that the presence of tBid may promote the formation of highly curved non-lamellar phases [57]. One surprising finding was the marked effect of procaspase-8 itself on the membrane and subsequent Bid binding. The additive effect of procaspase-8 and Bid may result from the acquisition of full functional activity upon binding to CL. The interaction of CL with caspase-8 on the membrane is important for the progression of apoptosis, with the formation of a local CL-protein reaction platform evident from the change in GP value. These results shed light on the role of mitochondrial membranes in the regulation of Bcl-2 protein family activity [33].

The results of rupture-tension experiments and those for Laurdan fluorescence are complementary. The rupture-tension approach, originally developed by Evans and coworkers [see [43] and citations therein], can be used to quantify the micro-mechanical properties of a thin film, such as a lipid membrane, by studying its deformation. Here, the expansion modulus (K_s_) and the rupture tension (τ_r_) were evaluated by expanding large unilamellar vesicles (GUVs), the membranes of which constituted a model system mimicking mitochondrial contact sites. We found that the addition of CL resulted in a marked decrease in the elastic moduli of DOPC lipid bilayers, with both K_s_ and τ_r_ strongly affected (**Fig. 3b and 3c**). The decrease in K_s_ following the addition of CL indicates that the membrane becomes easier to expand in the presence of this lipid (**Fig. 3b**). The CL molecule has an inherent conical shape; in a pure phase system, it would therefore preferentially be found in the inverted hexagonal phase. In the model system used here (CL/DOPC = 5/95 mol/mol), we intentionally avoided setting up such a condition: Each CL molecule was surrounded by DOPC molecules. In theory, the system displayed almost ideal mixing, as the chains of the two lipids (oleoyl-CL and DOPC) were identical. The predominance of the species preferring a lamellar phase ensured the maintenance of a lamellar state. The CL was, thus, structurally frustrated as it was embedded as a minor component within its host membrane. Nevertheless, its presence locally modifies spontaneous curvature. Due to their four hydrocarbon chains, CL molecules subjected to external force act like integrated springs that can be expanded more easily than the DOPC molecules, resulting in a lower K_s_. However, it is not possible for the system to assume a hexagonal phase and the limits of expansion of the lamellar phase are soon reached. The rupture tension is, therefore, lower than that for the pure system. The data for the pure control system are consistent with published data for DOPC vesicles [58], giving a K_s_ value of about 200 mN/m.

**Figure 6:**
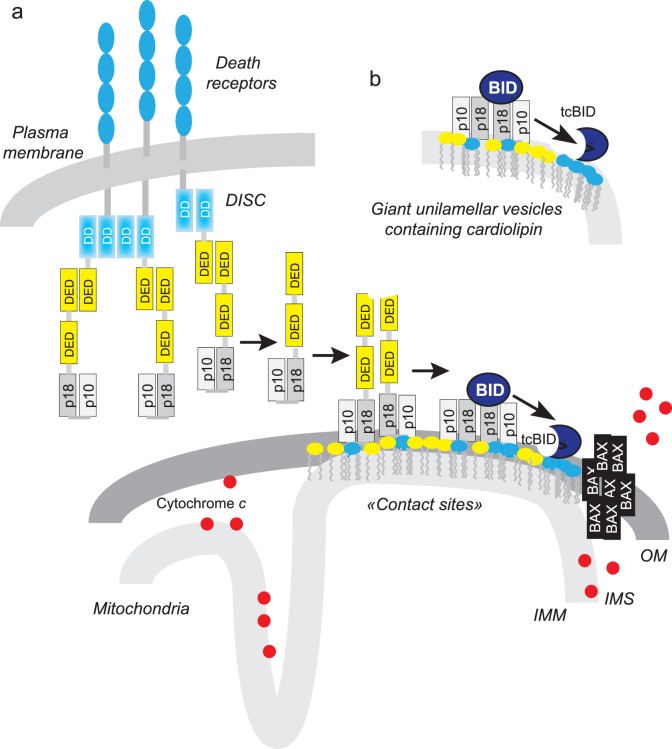
Localised production of active, cleaved BID on cardiolipin platforms that serve for the assembly of active caspase-8 and in the GUV “mimicking system”. (a) The diagram depicts the sequence of events in cells of type II according to Gonzalvez et al. [25]. The CL (red heads)/caspase-8 platform at the contact sites between inner and outer mitochondrial membranes (enriched in CL) binds BID resulting in the production of the active truncated, C-termimal part of BID (tcBID). This in turn causes CL induced perturbations of the membrane curvature, BAK/BAX oligomerization and cytochrome *c* release. (b) Schematic representation of the reconstituted functional platform on giant unilamellar vesicles containing CL with the p_18_/p_10_. DD, death domain; DED, death effector domain; p10 and p18 form the catalytic core of the caspase. The p43/p10 caspase-8 isoform comprises two DEDs, one p10 domain and one p18 domain. IMM, inner mitochondrial membrane; IMS, inter membrane space; OM, outer mitochondrial membrane. Red dots in the intermembrane espace, cytochrome *c* and the violet head correspond to the cardiolipin at the contact sites between outer and inner membrane.

We then assessed the mechanical consequences of proteins in the pure control system and in the PC/CL contact site model (**Fig. 3**). None of the proteins tested interacted readily with the pure DOPC control membrane. The properties of CL-containing GUVs were not changed by Bid binding, whereas the binding of caspase-8, tBid and caspase-8 plus Bid clearly modified the mechanical properties of these vesicles (**Fig. 3b and 3c**). The binding of caspase-8 alone partly reversed the effects of CL, indicating a role for CL in binding. The structural frustration observed when CL alone is added was reduced, such that the expansion module value was between those for the control and the DOPC/CL model system. The tensile breaking strength was essentially the same as that for the pure system, being limited only by the lipid membrane itself. Most probably, caspase-8 detects the curvature frustration close to CL locations within the membrane, and its insertion partially compensates for it. tBid alone also bound to the model vesicles (DOPC/CL). In this case, the expansive elastic response of the membrane, assessed by calculating the modulus K_s_, was fully restored to that of the pure DOPC control system: The adsorption of this protein fully released the structural frustration caused by the presence of CL. It is likely that all of the interaction sites were saturated. Nevertheless, the presence of the protein clearly caused defects that weakened the membrane to mechanical stress. This is evident from the very low value of the rupture tension. Although the membrane initially responded to a deformation force with an increase in area similar to that for the pure system, the total range of expandability was much lower, and the membrane broke down when the tension increased by ∼ 4.2 mN/m, corresponding to a change of ∼ 70% with respect to the control systems (pure DOPC or DOPC/caspase-8). Evidently, two domains with different elastic properties were formed. A major part of the membrane consists of essentially pure DOPC and does not participate in the interaction, or establishment of a reaction platform. Its elastic properties are therefore not modified, such that the observed K_s_ was ∼200 mN/m. The other part of the membrane, which contains CL as the initiator of a reaction platform, is more rigid. It does not discernibly contribute to membrane expansability but it limits the overall strength, as shown by the low value of τ_r_. A similar behaviour was observed for caspase-8 plus Bid, within the limits of experimental resolution, and in line with the GP results obtained with LUVs. All these findings are consistent with the recently described interactions between Bcl-XL [59] and tBid. We confirmed that CL plays an essential role in the association between caspase-8 and biomimetic membranes (**Fig. 6**), and most probably also biological membranes [25]. We suggest that CL is a component of the reaction platform formed subsequently (which also contain caspase-8 and Bid), in which it acts as a cofactor for caspase-8 activation. As the platform is formed, it immediately acquires its enzymatic function but only if CL is present (**Fig. 4** **and** **Fig. 5**). The production of tBid in the presence of caspase-8, when it interacts which CL, promotes vesicle breakdown; this effect is inhibited if caspase-8 inhibitors are added to the system [41]. These results indicate that the presence of caspase-8 linked to CL is essential for the formation of the so-called “mitosome” [25], [41]. In addition to interactions between CL and caspase-8, there may also be protein-protein interactions *in vivo*. It remains unclear whether other proteins, such as Rab5 [60], [61], which requires caspase-8 activation, or BAR [54] and FLASH, which mediate caspase-8 translocation to mitochondria [62], [63], [64], play an auxiliary role in the functional relationship between caspase-8 and CL. Possibly, MTCH2/MIMP [**65**] and its role in tBid recruitment may act in synergy with CL-induced mitosome formation to facilitate MOMP. The work we report here expands our knowledge of Bid-induced pro-apoptotic signalling and provides a description of the role of CL in capsase-8 recruitment and activation at the surface of the mitochondrial outer membrane. We are however far from grasping all the intricate and complex molecular alterations and interactions that lead to the activation of Bid, mitochondrial membrane permeabilisation and apoptosis via the mitochondrial pathway following stimulation of the death receptors.

The results that we present demonstrate and describe essential roles played by lipids in biological processes. In particular, they provide new insights into how mitochondrial specific lipids like CL can have active functions that go far beyond simply constituting a matrix for protein activities. Indeed, functional lipids appear to contribute not only to modulating the interactions between Bcl-2 family members, but also as key players in recognition processes as demonstrated by the example cardiolipin triggering the activation of caspase-8 in the apoptotic process.
